# Extracting salient sublexical units from written texts: “Emophon,” a corpus-based approach to phonological iconicity

**DOI:** 10.3389/fpsyg.2013.00654

**Published:** 2013-10-01

**Authors:** Arash Aryani, Arthur M. Jacobs, Markus Conrad

**Affiliations:** ^1^Department of Experimental and Neurocognitive Psychology, Freie Universität BerlinBerlin, Germany; ^2^Cluster of Excellence “Languages of Emotion,” Freie Universität BerlinBerlin, Germany; ^3^Dahlem Institute for Neuroimaging of Emotion (DINE)Berlin, Germany; ^4^Department of Cognitive Neuroscience and Psycholinguistics, University of La LagunaLa Laguna, Spain

**Keywords:** phonological iconicity, sound symbolism, foregrounding, text analysis tool, neurocognitive poetics

## Abstract

A growing body of literature in psychology, linguistics, and the neurosciences has paid increasing attention to the understanding of the relationships between phonological representations of words and their meaning: a phenomenon also known as phonological iconicity. In this article, we investigate how a text's intended emotional meaning, particularly in literature and poetry, may be reflected at the level of sublexical phonological salience and the use of foregrounded elements. To extract such elements from a given text, we developed a probabilistic model to predict the exceeding of a confidence interval for specific sublexical units concerning their frequency of occurrence within a given text contrasted with a reference linguistic corpus for the German language. Implementing this model in a computational application, we provide a text analysis tool which automatically delivers information about sublexical phonological salience allowing researchers, inter alia, to investigate effects of the sublexical emotional tone of texts based on current findings on phonological iconicity.

## Introduction

How do literary texts affect the reader? Can sounds have intrinsic, autonomous meaning, particularly in literary and poetic language?

The Russian Formalists were the first to examine these questions in a systematic fashion. They took the position that the phonological structure of poetry has a function beyond the decorative, and should be an object of study in its own right. In their examination of sound in poetry, the Formalists went far toward answering the questions of how sound patterns are organized in verse and what onomatopoetic types of sound patterns may be identified. Their approach provided a procedural basis for subsequent investigations of sound in poetry using objective criteria and a linguistic focus (Trotsky, 1957; Jakobson, 1960; Shklovsky, 1990; see Mandelker, 1983 for a review).

Over the last two decades, the study of the aestheticized form of language (e.g., literature, poetry) has been complemented by developments in cognitive linguistics, and particularly by research into an area at the interface between linguistics, literary studies, and cognitive psychology known as “cognitive poetics” (e.g., Tsur, 1992; Miall and Kuiken, 1994; Stockwell, 2002; Gavins and Gerard, 2003), “cognitive stylistics” (e.g., Semino and Culpeper, 2002; Semino, 2009), and “neurocognitive poetics” (e.g., Jacobs, 2011, 2013, 2014). This interdisciplinary field offers (neuro-) cognitive hypotheses to relate “the specific effects of poetry” to “the particular regularities that occur in literary texts” in a systematic way (Tsur, 2003). In contrast to other areas of literary studies, (neuro-)cognitive poetics deals with the central question of how readers comprehend and interpret the language of literary texts by conducting experiments “with different types of literary discourse, in different reading contexts with different kinds of readers” (Van Dijk, 1979).

In this article, we call upon the theoretical framework of (neuro-)cognitive poetics, particularly those insights emphasizing the figurative and embodied nature of linguistic knowledge, in order to investigate the relationship between the linguistic form of a text and the part of its meaning or emotional content, termed “iconicity”. We discuss the feasibility of iconicity being a potential indicator of the emotive qualities of a literary text specifically as evoked by particular phonemic structures. The basic idea behind such emotional impact of a literary text lies in the perceived similarity between the semantic content of the text and iconic associations based on the phonological salience at the level of the whole text as assumed by foregrounding theory (e.g., Garvin, 1964; Van Peer, 1986; Miall and Kuiken, 1994; Hakemulder, 2004).

Our measure of phonological salience is based on the deviation of the observed frequency from the expected frequency of particular phonemes or phonemic clusters in a given text. It's worth noting that the term “salience” used in this study refers to a technical term from literary analysis, and indicates an over-proportionally use of specific elements in a given text. We do not claim that our definition of the term “salience” always provides a perfect match to psychological salience in terms of how readers' perception of literary text might be affected by salient items. But still, our statistical operationalization might provide a good proxy for it in most cases. The focus of this study is on reliably extracting such phonologically salient units from written texts as a quantitative method facilitating further investigations in (neuro-)cognitive poetics.

### Foregrounding as a stylistic strategy

Foregrounding refers to a form of textual patterning and is capable of working at any level of language such as phonology, morphology, syntax, and semantics. It is essentially used as a stylistic technique to—from Shklovsky's Russian term *ostranenie*—defamiliarize the reading experience in textual composition through stylistic distortion of some sort, either through an aspect of the text which deviates from a linguistic norm or, alternatively, where an aspect of the text is brought to the fore through repetition or parallelism (Simpson, 2004). The analogy or correlation between the Gestalt notion of figure-ground (Rubin, 1921; Köhler, 1929; Koffka, 1935) and the idea of foregrounding theory in literature has led to applying the Gestalt principles to literary studies. The concept of figure-ground perception in Gestalt psychology is a fundamental organizing principle assuming that the perceiver distinguishes between incoming visual sensations and notices some as “more salient figures” in front of a “less salient ground” (Wallace, 1982). Figures, in literary texts, could be observed and classified in the sense of deviant or foregrounded features of literary language (Leech, 2008). However, only after empirical results have shown that literary foregrounded elements are related to a more intensive and extensive cognitive processing (Van Peer, 1986), increased memory for stylistic features (Zwaan, 1993) and deeper emotional experience (Miall and Kuiken, 1994; Hakemulder, 2004), the concept of foregrounding in modern text processing psychology has been seen “as a manifestation of the Gestalt psychological principle of figure-ground discrimination” (van Holt and Groeben, 2005). Besides empirical evidence from behavioral studies, recent functional neuroimaging experiments have provided supportive evidence for foregrounding theory and have shown that foregrounded items in a text can set the reader into a mode of aesthetic perception (Bohrn et al., 2012a,b, 2013). Comparing familiar with defamiliarized proverbs, Bohrn et al. have shown that defamiliarized proverbs increase cognitive processing demand and activate brain areas related to affective processing (orbitofrontal cortex, amygdala).

At the phonological level, foregrounded elements such as alliteration or rhyme can cause a “conscious sub-vocalization” and, at the same time, “aesthetic feelings, interest, curiosity, pleasure and self-reflection” (Jacobs, 2011). By means of four studies, Miall and Kuiken (1994) collected segment by segment reading times and ratings from readers of three different short stories which contained a variety of foregrounded features (i.e., phonological, grammatical, and semantic). They showed that the degree to which foregrounding is present in the segments of a story is a predictor of both reading times and readers' judgments of “strikingness” and “affect” and, interestingly, that the effective foregrounded elements were primarily phonological and semantic.

This assumed enhancing aesthetic and emotional effect of foregrounding on reading experience, combined with the semiotic notion of iconicity, which is discussed in the next section, will provide a theoretical framework to allow the computation of (sublexical) emotive qualities of a literary text by means of phonological foregrounded elements and their iconic associations.

### Iconicity in language and literature

In his semiotic theory of the sign, Peirce (1931) argued that the sign (representamen) can refer to its object through relationships of *similarity*, *contextual contiguity*, or *law*. According to this trichotomy, the sign is called an *icon*, an *index* or a *symbol* respectively. An Icon, or what is also called “a motivated sign” (Nöth, 2001), exhibits some relevant properties of the object; its form is similar to, and shares qualities with its referent. According to Peirce, there are three categories of iconicity: imaginal, diagrammatic, and metaphoric iconicity, with an increasing degree of abstraction related to their representing object. A well-known form of imaginal iconicity is the use of onomatopoeia, in which the referent is an acoustic signal and the link between sign and object is direct. In metaphoric iconicity, however, this link is indirect and associative (De Cuypere, 2008), i.e., a sensation, a feeling (e.g., happiness) or a property (e.g., size, color). The psychological aspect of the process of language comprehension, in the iconic case, “can be conceived of as the vicarious experiencing of events in the real world” (Segal, 1995; Zwaan et al., 2001; Zwaan and Yaxley, 2003).

By applying Peirce's iconicity to literary studies and poetry, Jakobson (1965) added instances of iconicity in syntax, phonology and morphology, and exemplified the similarity between form and meaning by the famous Caesar's phrase, “veni, vidi, vici” which mirrors “the order of narrated events in time or in rank” by means of syntactical and phonological icons such as cadence and repeating vowel sounds of the words. In his model of the functions of language, Jakobson distinguishes six elements or factors that are necessary for communication to occur. In poetry, according to this model, the emphasis is on the form of the message itself, and language not only serves as a medium of expression but is also “set toward the message as such, focus on the message for its own sake” (Jakobson, 1960). That is, the *differentia specifica* of poetry is located in its formal characteristics and iconic properties.

In the definition of the arts by Langer (1953) as “the semblance of felt life,” the relation of “form” to “feeling” lies at the basis of poetry and all art. This view has been expanded by Freeman (2009) who suggests that “form,” “feeling” and even “meaning” are all intertwined components of the cognitive processes of the embodied human mind in language and literature. Iconicity is an instructive example for the integration of these components in language—it creates “sensations, emotions and images that enable the mind to encounter them as phenomenally real” (Freeman, 2009). Lakoff and Turner, consistently, commented on the role of iconicity as “metaphorical image-mapping in which the structure of the meaning is understood in terms of the structure of the form of the language presenting the meaning” (Lakoff and Turner, 1989). Such image-mapping, according to them, is enabled by image-schemas which are formed from our embodied interactions. This embodied perspective of nature of icons, as a part of linguistic signs, challenges the simplified notion of the arbitrariness of linguistic signs.

### Linguistic signs: embodied or symbolic?

As language has been commonly considered symbol system in which meaning is constructed through formal symbol manipulation, the following question arises: how can purely arbitrary symbols operate with non-arbitrary embodied signs (e.g., icons)? The poetic use of language might be the best example of the convergence of two seemingly contrasting approaches to language comprehension; one a purely “symbolic” approach emphasizing the computational nature of symbols, and the other an “embodied” approach postulating the grounding of symbols in the physical world (Glenberg, 1997; Barsalou, 1999; Pulvermüller, 1999). But for all that, is language comprehension either solely symbolic or solely embodied?

Based on Peirce's trichotomy (icons, indices, symbols), Deacon (1997) suggests a hierarchical relationship of signs and argues that each sign is built from combinations of signs represented at its lower level (e.g., indices can be built from icons, symbols from indices). He claims that signs have the aptitude of operating not only with the signs in their own level but with those from other levels. Deacon proposes that the evolutionary jump to using a symbolic system from an iconic and indexical system and the ability to make links between symbols as well as between symbols and indices and icons can explain why humans have language (Deacon, 1997). This idea of a hierarchical relationship between signs is part of “the symbol interdependency hypothesis” which attempts to reach an agreement between two apparently contrasting accounts of language comprehension: symbolic and embodied (Louwerse, 2007, 2011; Louwerse and Jeuniaux, 2008). According to the symbol interdependency hypothesis, language comprehension is both symbolic and embodied. It can be symbolic by bootstrapping meaning through relations between the symbols, but it can also be embodied through the dependencies of symbols on indices and icons. This means that language comprehension is not solely symbolic because comprehenders can always activate embodied representations (e.g., indices and icons). The basic idea of the symbol interdependency hypothesis can be tracked back to Jakobson's view of the interrelatedness of sound and meaning. Jakobson supported a similar position to iconic and indexical use of language and proposed that “the iconic and indexical constituents of verbal symbols have too often remained underestimated or even disregarded; on the other hand, the predominantly symbolic character of language and its subsequent cardinal difference from the other, chiefly indexical or iconic, sets of signs likewise await due consideration in modern linguistic methodology” (Jakobson, 1965).

In the next section we will briefly outline some supportive results from studies in experimental psychology and linguistics on iconicity to account for its feasibility for extracting the emotional tone of a given text. Our focus will be on the relationship between the sound of words (phonological representations) and their meaning or emotional content; a phenomenon known as phonological iconicity that challenges the principle of arbitrariness—one of the tenets of Ferdinand de Saussure's theory of linguistic signs (De Saussure, 1916/1983).

### Studies in phonological iconicity

Besides onomatopoetic words, which sound like the concept they describe (e.g., click, bang, splash, boom), phonaesthemes are an instructive example for the relationship between sound and meaning (Schrott and Jacobs, 2011). Phonaesthemes are sounds, sound clusters, or sound types that are directly associated with a lexical category or meaning. The initial cluster /gl/ is often cited as an example of an English phonaestheme. It occurs in many words used for “shiny things”: glisten, gleam, glint, glare, glam, glimmer, glaze, glass, glitz, gloss, glory, glow, and glitter (Wallis, 1699; Magnus, 2001; Bergen, 2004). Likewise, in German, nouns starting with /kno/ and /knö/ are mostly small and round: Knoblauch “garlic,” Knöchel “ankle,” Knödel “dumpling,” Knolle “bulb,” Knopf “button,” Knorren “knot (in a tree),” Knospe “bud,” Knoten “knot (in string or rope)” (“Sound Symbolism,” n.d.).

The idea of phonological iconicity has ancient roots. In Cratylus (Plato, 1892), Plato has Socrates propose that the foundation of word semantics must lie in phonology and the way they sound. In contrast to de Saussure and Hjelmslev, many influential linguists supported the position of possible synchronic and productive effects of a word's sound on its meaning. For instance, Jakobson proposed that, “the intimacy of connection between the sounds and the meaning of a word gives rise to the desire of speakers to add an internal relation to the external relation, resemblance to contiguity, to complement the signified by a rudimentary image” (Jakobson, 1937). That is, the effect of sound in the mind completes its meaning and this, according to Jespersen, may lead to a kind of “natural selection” that “makes some words more fit to survive” (Jespersen, 1922).

Since the 1920's, there has been a considerable amount of increasingly sophisticated experiments to test the functioning of sound symbolism in languages. Sapir (1929), for example, raised the issue whether phonemes in isolation are symbolic of differing size by using two nonsense words MAL and MIL. Subjects consistently judged MIL to denote a small object and MAL to a large object. Building on this study, Newman (1933) further investigated the symbolic connotations of nonsense words differing (by pairs) only in one vowel and asked the subjects, which of the pair seemed larger, smaller, darker, or lighter. He concluded that tongue position in articulation is basic in the patterning of both magnitude and brightness categories.

More recently, a growing amount of literature has paid significant attention to understanding the role of phonological iconicity in language. Nygaard et al. (2009a) have reported that when native English speakers were presented with unfamiliar Japanese words, their ability to link these words to English meanings was biased due to sound-meaning relationships of Japanese words (see also Nygaard et al., 2009b). In a series of computational investigations, artificial language learning studies, and corpus analyses of English and French, Monaghan, Christiansen, and Fitneva have investigated arbitrary and systematic mappings between word forms and word meanings with regard to their respective advantages of word learning (Monaghan et al., 2011). They have shown that systematicity facilitates learning to group words into categories whereas arbitrariness facilitates learning specific word meanings (see also Christiansen and Chater, 2008). Several studies from experimental psychology and linguistics have suggested the existence of a systematic relationship between sound and meaning (Berlin, 1994; Cassidy et al., 1999; Bergen, 2004; Westbury, 2005; Farmer et al., 2006; Otis and Sagi, 2008; see Perniss et al., 2010 for a review). A considerable demonstration for a non-complete arbitrariness between sound and meaning is that both adults and infants map nonsense words with rounded vowels (e.g., bouba) to rounded shapes and nonsense words with unrounded vowels (e.g., kiki) to angular shapes (Köhler, 1947; Ramachandran and Hubbard, 2001; Maurer et al., 2006; Ozturk et al., 2013).

Apart from this general sound-meaning relationship, studies have explored a relationship between the way words sound and the emotional content expressed by them. Fonagy (1961), for example, applied statistical methods to examine the different tone qualities in six aggressive and six tender poems by the Hungarian poet Petöfi. While /t/, /k/, and /r/ were more frequent in aggressive poems, /l/, /m/, and /n/ were more frequent in tender poems. Based on information theory, Bailey (1971) analyzed a group of texts (poems vs. prose) to show whether in certain literary texts the frequency of occurrence of some phonemes is higher than this frequency in prose text, and revealed some meaningful patterns such as a preference for voiced over voiceless consonants and a preference for back over front vowels related to the aesthetic quality of poems. Recent machine learning experiments have shown that words expressing the same emotion (e.g., happiness) have significantly more in common with each other than with words expressing other emotions (e.g., sadness), suggesting that happy words might indeed sound happy (Nastase et al., 2007). In a similar study on affective words in Romanian and Russian, Sokolova and Bobicev (2009) captured the word form similarity by classification of words according to their emotion tags. The results suggest that the form of words allows for a reliable classification of emotion.

Evidence for the psychological reality of this sound-meaning correspondence comes from behavioral experiments. Wiseman and van Peer (2003), for instance, revealed that when German and Brazilian participants were asked to produce fantasy words corresponding either to the emotions experienced at a wedding or at a funeral, they tended to use similar consonants for respective emotional states, and this was independent of their native language. While nasal sounds (/m/, /n/) were more frequently used for the expression of sadness (funeral), plosive sounds (/p/, /b/, /d/, and /t/) were better suited to the expression of happy feelings (wedding) (Wiseman and van Peer, 2003; see Auracher et al., 2011). In an attempt to examine the universality of this phenomenon, Auracher et al. (2011) asked German, Chinese, Russian and Ukrainian native speakers to assess the emotional tone of poems with extreme values (highest vs. lowest) concerning the ratio of plosive vs. nasal sounds. Their results were consistent with the results of Wiseman and van Peer (2003) and confirmed the relationship between the emotional tone (happy vs. sad) of poems and the manner of articulation (plosive vs. nasal) for all examined languages. More recently, Myers-Schulz et al. (2013) have shown that the perceived emotional valence of certain phoneme combinations depends on the dynamic shift within the phonemes' first two frequency components, suggesting that certain strings of phonemes have a non-arbitrary emotional quality (e.g., /sa:/ is perceived as positive, and /za:/ as negative). In several analyses of English poetry and lyrics, Whissell has proposed that most of the basic sounds of English have emotional connotations attached to them (see, for instance, Whissell, 2003). She used the “*Dictionary of Affect in Language*” (Whissell, 1989) to validate these connotations and reported that, for example, the /l/ sound has positive and gentle connotations while /r/ and /g/ sounds have harsher ones.

### Extracting the salient phonological units

In sum, the use of stylistic elements in a text, such as foregrounded phonological salience can enhance aesthetic and emotional effects on reading experience. On the other hand, the perceived similarity or analogy between the phonological forms of those salient elements and their meaning, or their emotional impacts on the reader, as assumed by the semiotic notion of phonological iconicity, can be a reliable source to predict a part of the emotional and aesthetic qualities of a given text. Now, what exactly makes a phoneme or any other sublexical unit within a given text, regardless of its genre, “salient”?

All the above mentioned theoretical claims or empirical approaches to phonological iconicity encounter the following problem: They assume that single sublexical units already relate to semantics below the lexical level in that they either possess an iconic value on their own, or directly convey meaning via a basic grounding of semantics or emotion at the level of single sounds. But unlike words, all these more basic elements supposed to carry such basic semantic or emotional “meaning” almost necessarily have to form part of any complex speech signal—given the general limitations of the phoneme inventory of natural languages. Thus, how could a single sublexical unit serve as a linguistic sign conveying specific semantic meaning if it almost necessarily will be present in any text, regardless of its meaning? A plausible tentative answer to this question is: frequency.

Single sublexical units only gain such “semantic sign” character if occurring with elevated frequency within a given text. Notably, this is the point of view adopted by approaches trying to assign emotional values to sublexical units on a purely empirical base (e.g., Bailey, 1971; Whissell, 2003, 2009; Auracher et al., 2011) establishing relationships between lexico-semantic word meaning and sublexical organization. But what kind of information regarding the frequency of occurrence of single sublexical units could be extracted from single texts standing in the focus of interest?, as this would be the case for all kinds of scientific approaches focusing on already existing language samples as literary productions. Here, the analysis of frequencies of occurrence for single sublexical units—in order to label them as “salient” or not—faces two issues:

– The more trivial one of how to count them?
– The less trivial one of when to label a given frequency of occurrence as salient?

## Materials and methods

To extract the salient sublexical units within a text, we developed a probabilistic model. The basic idea is to weight the frequencies of occurrences of a sublexical unit in a text by comparison to a linguistic corpus serving as a reference. Both single phonemes and sub-syllabic segments (e.g., onset, nucleus, and coda) have been considered as relevant indications of phonological iconicity. The higher the discrepancy between the relative occurrences of sublexical units within a given text on the one hand and within the reference corpus on the other, the more “significant” the specific use of such units may appear.

According to foregrounding theory, the foregrounded pattern in a literary text or in poetry deviates from a norm, either through replication or through parallelism. In his neurocognitive model of literary reading, Jacobs (2011, 2013) assumes that not the deviation by itself, but the ratio between deviation and standard, between foregrounded and backgrounded elements, is the crucial factor for emotional and aesthetic experiences of literary reading. To proceed on the assumption that these norms originate from everyday spoken language, a corpus that is to be used as a reference should be representative of everyday language.

### Reference corpus

Recent studies have shown that word frequency calculated from corpora based on films and television subtitles can better account for reading performance than the traditional word frequency based on books and newspapers, since the language used in subtitles greatly approximates everyday language (interactions with objects and other people); evidence comes from: German (Brysbaert et al., 2011), Spanish (Vega et al., 2011; see also Duchon et al., 2013), Greek (Dimitropoulou et al., 2010), Chinese (Cai and Brysbaert, 2010), and Dutch (Keuleers et al., 2010). Brysbaert et al. have shown that frequency measures should best be based on a corpus of at least 20 million words (Brysbaert et al., 2011). They reported that the best quality of frequency measures for German regarding their correlation with behavioral word processing data is observed for SUBTLEX-DE; a corpus of 25 Million German words consisting of movie and television subtitles. Accordingly, we decided in favor of SUBTLEX-DE as a reference corpus.

As the idea of phonological iconicity takes account of the phonological feature of language rather than orthography, each text to be analyzed should be converted into phonetic notation before analyzing it. For this purpose, we chose the German text-to-speech synthesis system MARY (Schröder and Trouvain, 2003), which has a modular design that allows the user to access the grapheme-to-phoneme part separately. MARY uses the SAMPA phonetic alphabet for German (Wells, 1997) and outputs the phonemic transcription in an internal, low-level markup language called MARY-XML. To phonemize the text automatically, an extensive lexicon deals with known words and a letter-to-sound conversion algorithm with unknown words.

Since the selected corpus SUBTLEX-DE is only available in orthographic form, it was necessary to translate also the whole reference corpus into phonemic notation. On that account, we have split the corpus into small units, each including approximately 30000 characters^1^. Translating more than 6000 units through MARY, parsing resulted XMLs and then integrating them in one dataset, we have generated a complete phonemized corpus for the German language; a useful resource for future research in the area of psycholinguistics.

**Figure 1:**
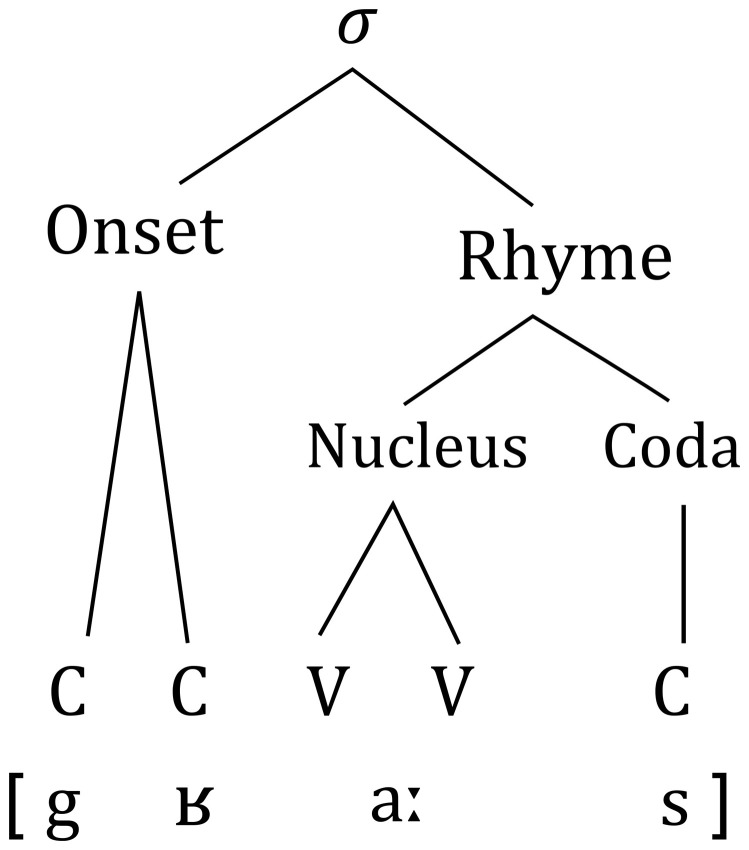
**Representation of the structure of a syllable (σ) using the example of the German word “Gras”**.

The following steps of analyses will focus on relative syllabic position of phonemes. Since syllable separation for each word is marked in phonemized SUBTLEX it was possible to extract all syllables of existing words. Based on internal structure of a syllable (Figure 1) and a list of all 19 vowels in German (16 monophthongs and 3 diphthongs) each sub-syllabic unit has been segmented and its frequency of occurrence in the corpus has been counted.

Additionally, the sum of all sub-syllabic units and all phonemes existing in the corpus have been calculated in order to obtain the relative frequency of occurrence of each unit (Table 1).

**Table 1:** **List of 10 most frequent phonemes and sub-syllabic units in SUBTLEX-DE (in SAMPA alphabet)**.

**Phoneme**	**Freq (%)**	**Onset**	**Freq (%)**	**Nucleus**	**Freq (%)**	**Coda**	**Freq (%)**
n	9.608	d	13.431	@	17.462	n	26.010
t	7.282	v	7.965	I	13.288	6	13.266
@	6.714	n	7.863	a	11.199	s	10.519
I	5.110	z	7.262	i:	8.667	C	7.894
6	4.761	t	6.792	E	7.637	t	4.930
s	4.535	m	6.792	aI	5.863	l	4.168
d	4.351	g	5.572	a:	5.467	st	3.793
a	4.306	b	5.358	e:	4.684	m	3.508
l	3.372	f	4.891	u:	4.023	nt	3.464
i:	3.332	h	4.756	U	3.947	Ct	2.378

### The probabilistic model

Since the significance of the use of a certain sub-syllabic unit depends on both its relative frequency of occurrence and the length of a given text, the intended model has to be able to predict the expected frequency and its standard deviation according to the corpus as a function of relative frequency and text length. Given the frequency of occurrence of a certain sub-syllabic unit in the corpus *f*_*u*, *c*_, the text length *n*_text_, and the corpus' length *n*_c_, the expected frequency of occurrence of this certain unit in a given text would be:

(1) $$E_{u,\text{text}}=f_{u,c}\cdot \frac{n_{\text{text}}}{n_{c}}$$

For simplification, the term *f*_*u*, *c*_/*n*_*c*_, which is the relative frequency occurrence of a certain sub-syllabic unit will henceforth be called as *f*_*u*, *r*_.

**Figure 2:**
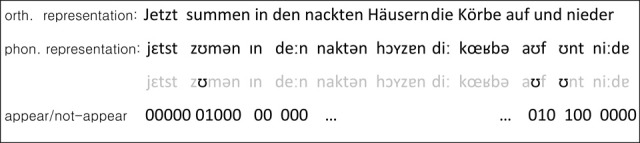
**A random text is simulated as random binary sequences 000000100 …**. The symbol “1” appears with probability *p* and models a successful occurrence of a certain sub-syllabic unit in a text (in this example the phoneme ℧), and the symbol “0” accounts for its unsuccessful occurrence with probability 1*-p*.

For calculating the standard deviation as a function of *n*_text_ and *f*_*u*, *r*_, one approach consists of considering the occurrence of each sub-syllabic unit in each position of the text as a success/failure experiment with a certain probability. That is, we consider the whole text as a collection of gaps which could be filled by any sub-syllabic units contributing to the formation of the whole text. From this perspective, each given text could be seen as a sequence of *n*_text_ × yes/no experiments regarding each particular sub-syllabic unit; as if all units were competing for filling each gap with a specific probability (Figure 2).

Assuming that the occurrence of each unit in each position in the text is independent of the other units (which is not necessarily given for language) and that the probability that it succeeds in filling the position is equal to its relative frequency, one can consider the text as several Bernoulli trials with a binomial distribution. The standard deviation of a binomial distribution is:

(2) $$u\sim \text{B}\left(n_{\text{text}},f_{u,r}\right)\to \sigma u=\surd n_{\text{text}}\times f_{u,r}\times \left(1-f_{u,r}\right)$$

**Figure 3:**
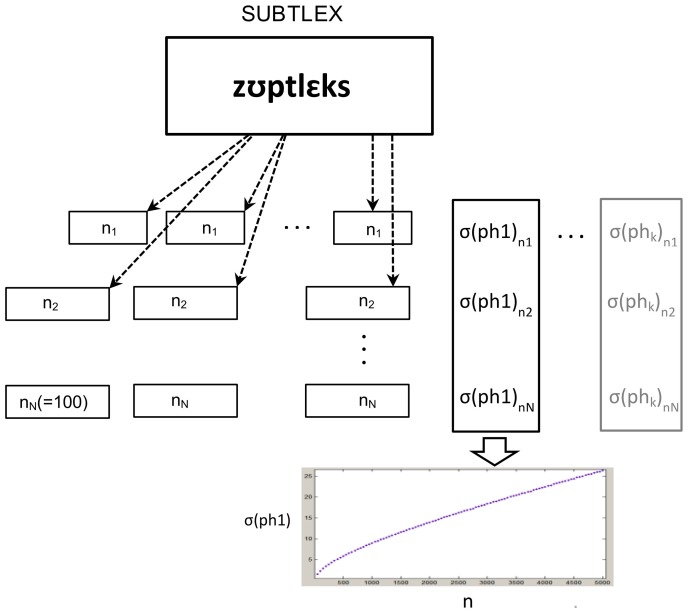
**Calculation of standard deviation for each set of pulling**. Note that the relative frequency of each sub-syllabic unit is constant giving one function for each unit.

Given the fact that only specific arrangements of sub-syllabic units can contribute to meaningful words, it is obvious that the distribution of sub-syllabic units in a text does not have completely random characteristics as preconditioned by a binomial distribution. However, this distribution can help to form a rough estimate about the characteristics of standard deviation and its dependency on both input factors (i.e., *n*_text_, *f*_*u*, *r*_). To calculate standard deviations more precisely, we chose an empirical approach by pulling numerous chunks of sub-syllabic units from the corpus. For a text with a certain length, a text sample with the same length is randomly pulled from the corpus and the frequency of occurrence of all sub-syllabic units is counted in this sample. Since the samples should be representative for a larger population, this procedure is repeated (with replacement) for 1 Million times for each specific length of text. It's worth pointing out that the corpus includes almost 25 Million words and more than 2 Million sentences. We opted to let all chunks start with the beginning of a sentence, because this best represents normal language. In consequence, 2 Million different chunks can be pulled from the corpus. For each set of pulling (i.e., 1 Million samples) the standard deviation for any sub-syllabic unit is calculated giving one point in the Cartesian coordinate which is representative for the value of standard deviation for a certain sub-syllabic unit dependent on the length of a given text (Figure 3).

**Figure 4:**
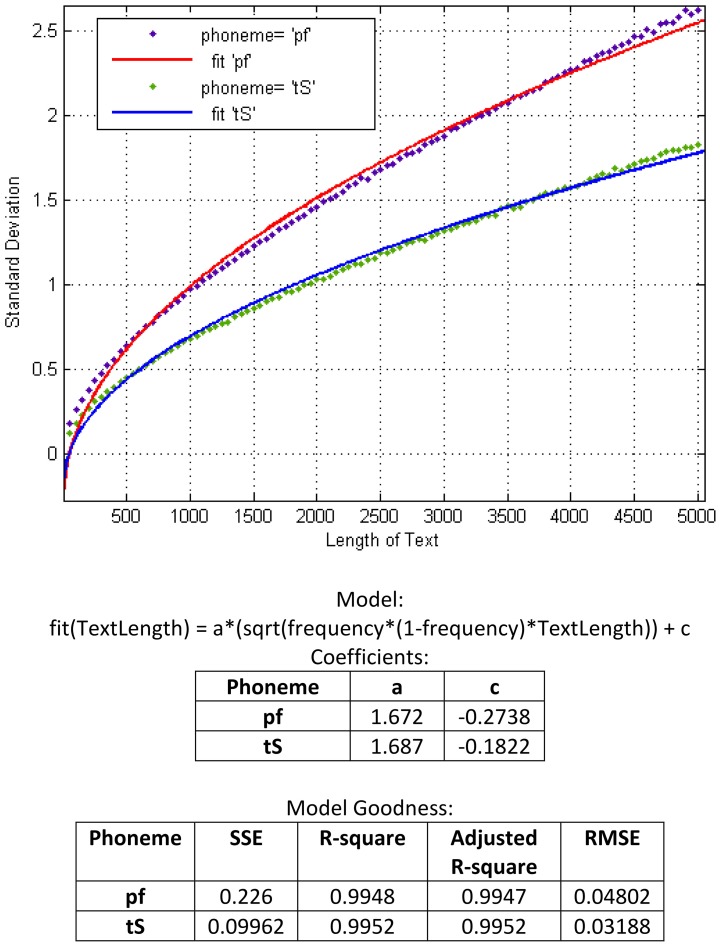
**Binomial-based model for prediction of standard deviation using the examples of “pf” and “tS”**.

This procedure of pulling is repeated for different lengths of texts, starting with the minimal size of 50 units for the first step, sampling 100 different lengths with steps of 50 units each (i.e., 50, 100, 150, …, 5000) and making separate measurements on each series of texts with a certain length (Figure 3). The analysis of all obtained curves shows, as expected, similar characteristics to a binomial distribution. Based on the mathematical equation for calculating the standard deviation of binomial distribution, we construct a curve that has the best fit to the series of our data points. In Figure 4 we represent standard deviation as a function of a text length using the example of the phonemes “pf” (as in the German word “Pferd”) and “tS” (as in the German word “Deutsch”).

**Figure 5:**
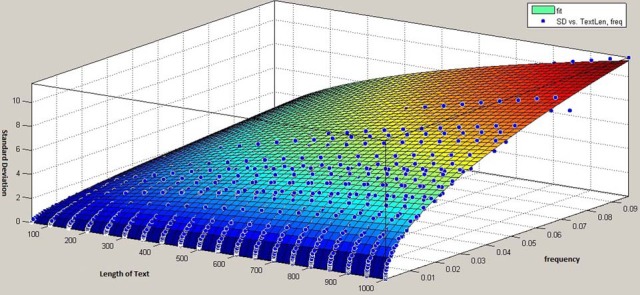
**The 3D-model for prediction of standard deviation of sublexical units as a function of text length and frequency**.

Note that the curves in Figure 4 represent standard deviation as a function of text length alone, and not of relative frequency. In fact, each phoneme (as each sub-syllabic unit) has a certain relative frequency, which means, the factor frequency doesn't appear as an input parameter, but rather as a constant value which is integrated in the function. In so doing, we could obtain a number of single functions each of which can predict the standard deviation of a certain sub-syllabic unit or a phoneme dependent on the text length. In order to obtain a general model for the prediction of any sub-syllabic units or phonemes, we compiled our all data in a three dimensional space as following: one dimension standing for the frequency of each sub-syllabic unit or phonemes (giving one point for each unit), one dimension standing for the text length (50, 100, 150, …, 5000), and one dimension for each standard deviation related to other two dimensions. In this three dimensional space and, again, based on binomial distribution, we constructed a surface for the best fit to our data (Figure 5).

Though the accuracy of this latter model is less than accuracy of single functions for each unit, having a general model with text length and frequency as inputs simplifies the determination of standard deviation.

It's worth mentioning that this empirical approach is not needed for determining of expected value, because the calculation of expected value, which is based on formula 1, does not assume a particular type of distribution as it was the case for the calculation of standard deviation.

### The text analysis tool “Emophon”

**Figure 6:**
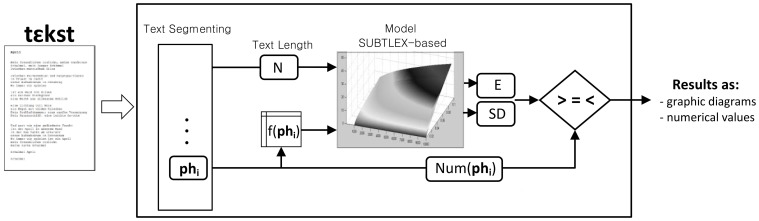
**Representation of workflow and modules employed in the tool**.

Based on the aforementioned model, we developed the computer linguistic tool Emophon which is a flexible tool for research and analysis of literary texts at the sublexical level. Emophon allows a step-by-step processing with an access to partial processing results. The core of the tool, as represented in Figure 6, consists of a segmenting module, the SUBTLEX-based model, and a comparing module. All sub-syllabic units and phonemes of a given text (phonemized through MARY) are found and segmented. By means of the frequency value of each unit (signed as f(Ph) in the representation) and the text length (calculated by tool), the integrated model provides a correspondent expected value and standard deviation for each unit. The number of each existing sub-syllabic unit and phoneme in the text is compared with the confidence interval provided by the model. Results are outputted as both graphic diagrams and numerical values of the degree to which the confidence interval is exceeded or not.

A Graphic User Interface (GUI) allows user to browse and select texts. The sublexical salience in the text can be extracted and shown for structures at the following levels by simply selecting the corresponding option given in the GUI:

– Whole phonemes in the text
–Sub-syllabic onsets
–Sub-syllabic nuclei
–Sub-syllabic codas
– All single phonemes appearing in onsets
–All single phonemes appearing in codas

Emophon has been completely written in “Python” (V2.5) and uses external libraries “numpy” and “mathplotpy.”

## Results

In the following we will first demonstrate and validate the functionality of Emophon and then show how it can be used as a linguistic instrument for analyzing and extracting the phonological salient units in a text.

### Validation of the functionality

**Figure 7:**
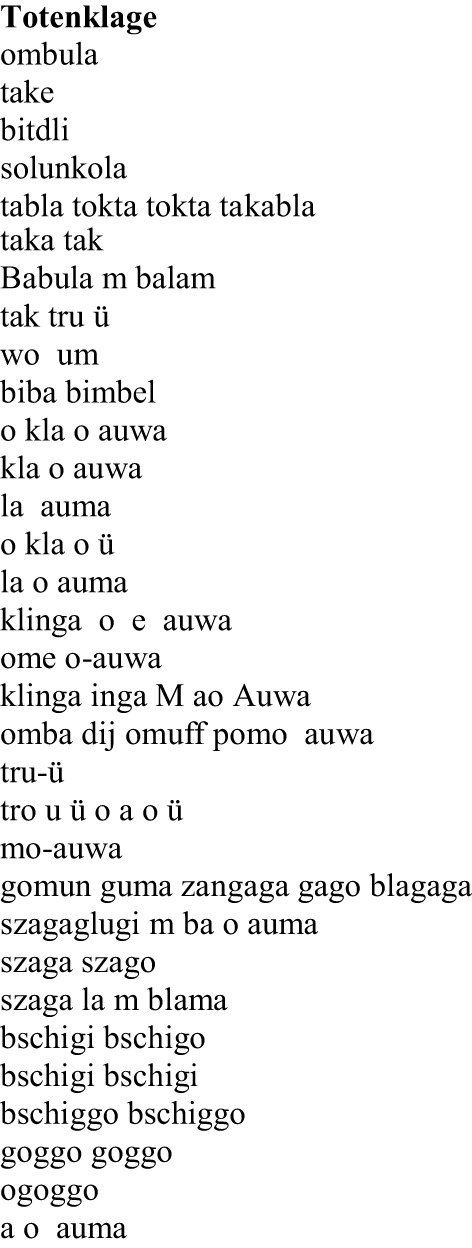
**Hugo Ball's poem “Totenklage”**.

To validate the functionality of Emophon we used a particular poem for which phonological deviation is known to some degree: the poem “Totenklage” written by German poet Ball (1916/1992); one of the leading poets of Dada movement and a pioneer of *sound poetry*. In sound poetry, phonological aspects of a poem are foregrounded. In this genre of poetry, linguistic meaning waives in whole or in substantial part and the language is purely formal and can be seen as mere sound material. The selected poem “Totenklage,” in particular, is characterized by the frequent use of word-like units consisting of open syllables (CV; otherwise rather untypical for the German language) using preferentially specific vowels (see Figure 7).

**Figure 8:**
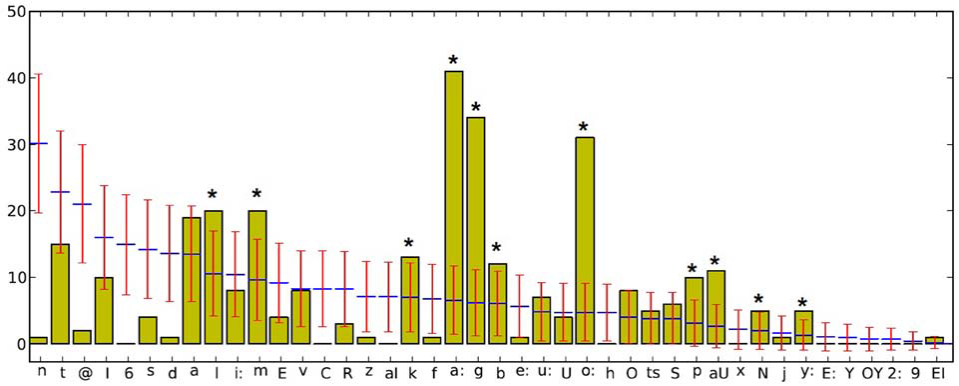
**Diagram of phonemes in the poem “Totenklage” as outputted by the tool**. Expected values of each phoneme are marked with the blue line, confidence intervals with red.

Since the “words” used in this poem are artificial, one might expect a high degree of phonological deviation from the German language and, in consequence, a large number of phonemes labeled as salient by the tool. In particular, we expect an exceeding of the confidence interval for the two vowels; /a:/ (as appeared in: tabla, tokta, taka, babula, biba, kla) and /o:/ (as appeared in: solunkola, wo, o, ome, omba, pomo) as well as for the consonant /g/ (as appeared in: gomun, guma, gago, goggo, bschiggo) because of their apparent high frequency of occurrence in the text. The poem was first converted to phonetic notation by using MARY. After reading of the phonemized text by the tool, one can optionally select one of the above-mentioned levels of seeking salience in the text (phonemes, onsets, nucleus, etc.). The graphical demonstration of results concerning the phoneme level is presented in Figure 8. The number of existing phonemes, the expected value and the confidence interval for each phoneme in the text (based on calculated standard deviation) are represented in the diagram. Phonemes that were significantly more frequent than expected and consequently exceeded the confidence interval are signed with an asterisk.

In addition, the exact degree to which each phoneme exceeds the confidence interval is outputted by the tool and represented in Table 2.

**Table 2:** **Numeric representation of the salient phonemes in the poem “Totenklage”**.

**Table of salient phonemes**			
**Phoneme**	**Tokens of this phoneme**	**Tokens of phonemes outside CI**	**Upper/Lower**
a:	41	29.32	Upper
g	34	22.87	Upper
o:	31	21.94	Upper
aU	11	5.13	Upper
m	20	4.26	Upper
p	10	3.39	Upper
l	20	3.02	Upper
y:	5	1.36	Upper
b	12	1.05	Upper
k	13	0.76	Upper
N	5	0.17	Upper
h	0	−0.40	Lower
f	1	−0.62	Lower
z	1	−0.84	Lower
aI	0	−1.81	Lower
C	0	−2.60	Lower
s	4	−2.85	Lower
d	1	−5.42	Lower
6	0	−7.39	Lower
@	2	−10.19	Lower
n	1	−18.70	Lower

Note that fractions appear since the value of confidence interval is calculated based on the mathematical model.

As predicted, all of three mentioned phonemes (i.e., /a:/, /o:/, /g/) were marked as salient and positioned in the top of the salient list (Table 2 and Figure 8). Notably, in addition, the phoneme /aU/ (as in the “words” “auwa” and “auma”) largely exceeds its respective confidence interval (5.13 out of 11) though its absolute number of occurrence in the text is rather low. This is due to the fact that the expected value and the confidence interval for each phoneme positively correlate with its relative frequency in the corpus, which is low in this case. Similarly, by comparing the statistical salience of the phonemes /n/ and /d/ (with identical frequencies of occurrence = 1), it becomes obvious that /n/, which has a higher relative frequency in the corpus and, accordingly, a higher expected value, is marked as more salient—due to its unexpected low actual occurrence in the text—than /d/ (18.7 for /n/ vs. 5.42 for /d/, less than expected). For the same reason, the phoneme /e:/, which has the same frequency of occurrence as /n/ and /d/ isn't marked by the tool as salient at all, by virtue of its very low relative frequency in corpus (Figure 8).

**Figure 9:**
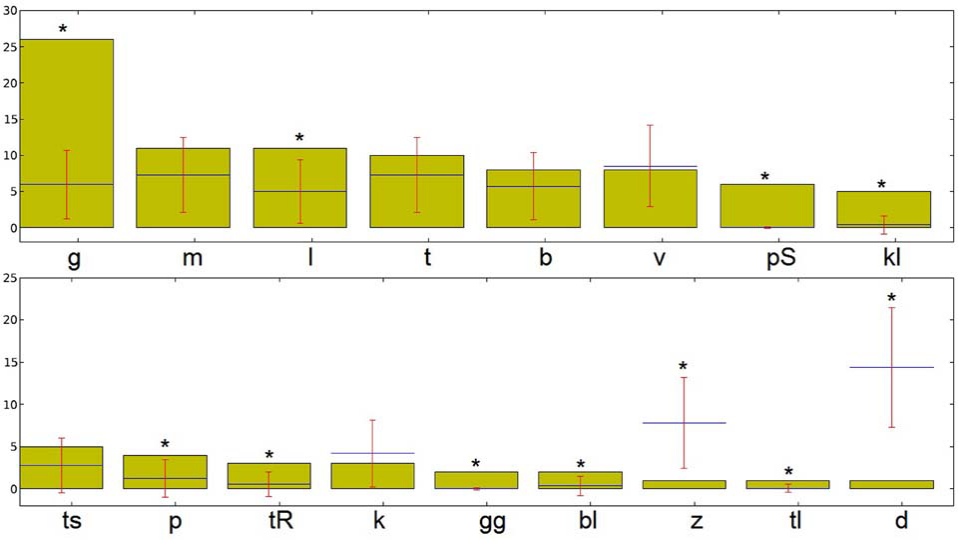
**Diagram of “salient” syllabic onsets in the poem “Totenklage” as outputted by the tool**. ^*^Denoting onsets that were significantly more (or less) frequent than expected.

Similar analyses can be conducted at the all above-mentioned levels to extract the salient sub-syllabic units in the text. Figure 9 demonstrates results of such an analysis for sub-syllabic onsets for the same poem. Similar to the previous analysis of phonemes, a large portion of existing onsets is marked as salient. In addition to the salient phoneme /g/ from the previous analysis, the onsets /pS/, /kl/, /bl/, and /tr/ show a large degree of salience in the poem (the exact numeric values of saliency are not shown due to the limited place).

### Testing an exemplary hypothesis

To illustrate the potential use of Emophon as a linguistic instrument for analyzing the phonological salient units in texts following an exemplary hypothesis, we focus on the theory of foregrounding assuming that poetic language deviates from norms characterizing ordinary language use. We hypothesize that this deviation is observable at the phonological level and that it can be measured by the numbers of salient phonological units as provided by our tool. To this end, we chose 20 classical poems of approximately equal length (number of characters with space) from different German poets. All these poems are characterized by the two classical patterns of metric alignment and syllabic rhymes at the end of two lines either following an AABB or an ABAB pattern. In particular, these syllabic rhymes should lead to an increase in frequencies of occurrence of syllabic nuclei and codas in these examples of lyrical language when compared to everyday common language use.

As control texts, we chose 20 text passages—matching the lyrical texts in length—that had appeared in different online German newspapers as respective first articles on the day of analysis (see Appendix for a complete list of poems and newspaper articles). The subjects of these articles vary topically ranging from political to local news. After converting all texts in phonetic notion and analyzing them by the tool, we documented phonological salient units at 4 different sublexical levels: at the level of (1) phonemes, (2) sub-syllabic onsets (3) sub-syllabic nuclei, and (4) sub-syllabic codas. For a statistical comparison between the two groups of texts, we defined two measures indicating the degree of phonological salience for each text: (1) the number of phonological units being salient (i.e., the number of rows in Table 2 for the previous text), and (2) the absolute sum of all segments positioned outside the confidence interval (i.e., the absolute sum of the numbers in the 3rd column of Table 2). Thus, we obtained 8 indicators for each text, all of which can serve to compare poems and newspaper articles. The values of these indicators for each text according to the tool performance are represented in Table 3.

**Table 3:** **Numeric representation of the sublexical salient units for all of 20 poems and newspaper texts**.

	**Phoneme**				**Onset**				**Nucleus**				**Coda**			
	**Num**		**Sum**		**Num**		**Sum**		**Num**		**Sum**		**Num**		**Sum**	
**Text**	**News**	**Poem**	**News**	**Poem**	**News**	**Poem**	**News**	**Poem**	**News**	**Poem**	**News**	**Poem**	**News**	**Poem**	**News**	**Poem**
1	3	6	3.56	8.39	11	12	6.00	11	3	3	2.58	2.71	8	13	4.93	14.76
2	7	9	14.76	22.01	9	11	8.83	13.42	3	6	8.19	9.70	10	11	10.42	10.57
3	5	4	5.45	20.67	3	11	8.43	20.14	2	2	1.03	2.45	10	9	13.36	9.06
4	5	6	5.45	10.47	3	9	8.43	5.67	2	3	1.03	7.17	10	6	13.36	14.22
5	4	7	14.04	12.50	9	4	8.45	3.44	2	6	10.71	6.84	12	8	10.59	6.26
6	9	7	17.26	13.97	17	9	17.32	6.38	4	4	7.74	7.33	7	8	6.06	9.95
7	5	10	17.79	15.89	9	5	18.71	6.40	2	4	4.61	12.2	9	15	13.10	20.56
8	3	6	3.83	9.43	5	5	6.26	8.15	3	3	3.20	1.75	3	12	4.68	9.87
9	9	6	20.95	20.21	7	6	12.10	6.46	3	6	8.41	21.06	7	7	6.06	2.33
10	5	10	10.31	29.45	9	9	12.82	8.05	0	6	0.00	24.69	6	10	2.69	16.43
11	2	3	1.10	9.46	5	6	4.34	8.76	0	4	0.00	11.3	9	10	5.37	5.44
12	8	8	24.98	11.39	11	8	18.53	5.56	5	3	9.55	7.39	10	7	4.96	3.44
13	8	7	24.19	14.66	11	9	14.02	19.91	4	1	12.26	1.41	3	11	6.78	11.08
14	8	9	12.89	12.22	7	10	8.53	7.49	5	4	10.06	5.53	8	12	9.44	11.28
15	7	8	13.75	15.36	9	7	6.57	12.75	2	3	8.96	7.23	3	11	6.01	7.57
16	5	9	15.23	14.84	6	4	4.03	4.10	2	4	10.35	14.76	6	10	10.54	23.32
17	4	10	3.11	32.75	4	8	2.37	12.72	2	7	2.92	18.21	8	9	8.67	10.13
18	6	9	8.86	26.62	10	11	8.44	12.13	2	6	4.88	13.07	8	5	6.34	14.76
19	4	9	11.62	19.89	6	11	2.43	9.05	2	3	7.09	8.95	8	8	4.34	5.85
20	1	8	2.27	10.47	9	3	7.69	1.99	0	4	0.00	8.95	2	11	1.92	13.72

“Num” stands for the number of distinct phonological units being salient (the 1st indicator) and “Sum” for the absolute sum of all segments positioned outside the confidence interval (the 2nd indicator).

Applying *t*-tests on these 8 indicators, we assessed the likelihood that the means for the two types of texts (poems vs. newspaper) are sampled from the same sampling distribution of means. The results revealed a significant effect of text's type on the number of salient phonemes, *t*_(38)_ = 3.18, *p* = 0.003, on the number of salient nuclei, *t*_(38)_ = 3.56, *p* < 0.001, and the number of salient codas, *t*_(38)_ = 2.76, *p* = 0.008, with each time more salient units being present in poems than in prose texts. Similarly to the number of salient units, there was a significant effect of text's type on the absolute sum of segments positioned outside the confidence interval: for phonemes, *t*_(38)_ = 2.2, *p* = 0.033, for nuclei, *t*_(38)_ = 2.35, *p* = 0.023, and for codas, *t*_(38)_ = 2.47, *p* = 0.017, again, with more salient segments in poems than in newspaper articles. The means of the number and the absolute sum of salient onsets did not significantly differ between two groups: *t*_(38)_ = 0.1, *p* = 0.91, and *t*_(38)_ = 0.02, *p* = 0.98, respectively.

These results are consistent with the theory of foregrounding at the phonological level. All poems used for the present analyses contain shared rhymes across endings of lines. Considering the internal structure of a syllable, where rhymes involve syllabic nuclei and codas, the significant higher mean of the number and the sum of salient nuclei and salient codas in poems as compared to prose texts reflects how such typical features of lyrical texts using foregrounding at a phonological level can emerge in the analyses conducted with our tool. When selecting texts for the present exemplary analyses, we focused on rhymes as the one lyrical feature to be compared between lyrical texts at the one hand and more prosaic everyday language use at the other. Meeting our hypothesis, results from the tool showed that in particular for those sub-syllabic segments that are essential for rhymes, syllabic nuclei, and codas, significant differences between the two groups of lyrical and non-lyrical texts were obtained.

But certainly, the use of other stylistic techniques and sound effects such as alliteration, consonance, assonance and in particular phonological iconicity in poetry or related literary genres, could obviously also be addressed by our tool once particular hypotheses have been formulated and adequate texts have been chosen for analyses. Results obtained for syllabic onsets from the poem Totenklage (see Figure 9) or the finding of significantly higher means of the number and the sum of salient phonemes in poems as compared to control texts might suffice as initial examples for the potential of such enterprises.

## Discussion

In this paper, we presented a computer-linguistic tool automatically transforming digitally presented text into phonetic transcriptions, the absolute frequencies of which are documented and compared to expectation values derived from a large scale database of 25 Million German words representing everyday language use.

The user of this tool is provided with the information of whether any specific sublexical unit occurs more or less often than this would be expected for exactly this specific unit given the length of the text it was taken from and, importantly, the corresponding degree of derivation from expectation values is an object of a statistical inference process assigning levels of significance for deviations based on a probabilistic model.

Our tool, thus, lays the ground for all kinds of empirical research relying on the assumption that what is generally understood as higher level intentional message or emotional tone of a text may already be observable at the sublexical level. Beyond providing a text analysis tool for establishing frequencies of occurrences of phonologically defined sublexical units of different grain size within texts, the novel contribution of our tool mainly consists in implementing a mathematical model which offers a straightforward way to transform the mere observation of these frequencies of occurrence into inference statistical conclusions regarding their significance—thus, resolving the problem of how to define and assess the salience of small units of language embedded in large texts.

Once the frequency of occurrence of a given phonological sublexical unit within a text is identified by the presented tool as possessing a salient status, it is reasonable to assume that the specific unit might be used in a given text as an element of foregrounding—especially in the artistic domain of language use where the connection between formal aspect of language and higher meaning is of great importance.

It's worth noting that we opted for a phonological definition of these potentially relevant foregrounded elements in texts, because most literature on correspondences between meaning and sublexical structures traditionally focuses on language's sound as the relevant perception domain. In consequence, all analyses of the present tool are based on phonologically described sublexical units. Of course, this does not preclude an additional, and potentially independent, role of specific visual features of words or letters as foregrounded elements with potential iconic status (see for instance Doyle and Bottomley, 2009, 2011). A differential examination of phonological vs. visual phenomena is, yet, beyond the scope of the present paper.

The sublexical salience fingerprint of written texts—provided by our tool—can then also be related to theories and approaches claiming a specific emotional value for single sounds or other sublexical phonological units in language. Once their salience has been established, the emotional status of such sublexical units—potentially being used as foregrounding elements in the given context—could then serve to predict the sublexical emotional tone of a given text as a function of the emotional status of salient sublexical units.

Such an enterprise may be useful for different types of texts and different research motivations ranging from general to applied linguistics or psychology. Its use may though, be especially evident in the case of lyrical language: For instance, the emotional tone of the poem “Totenklage” (the first poem reported in this article) might be predicted by focusing on salient vowels in this text as following. The “frequency code” theory by Ohala (1994, 1996) posits sound-meaning correlations in intonational communication of affect and in iconic vocabulary: whereas high pitch sounds signify smallness, non-threatening attitude, desire for goodwill of the receiver, and generally positive emotional valence, low pitch sounds convey largeness, threat, self-sufficiency, and generally negative emotional valence. Regarding this categorization, vowels with lower pitch (e.g. /u/, /o/, /a/) might be associated with negative emotion and vowels with higher pitch (e.g. /i/, /e/, /@/ and to some extent /6/) with positive emotion. Considering the extracted salient vowels in Hugo Ball's “Totenklage,” the emotional tone of this poem at sublexical level (based on Ohala's theory) would be revealed as negative: where /o:/ and /a:/ appear significantly more than expected, the poem includes /@/ and /6/ significantly less than it was expected. This finding seems perfectly consistent with poet's presumable intent as evidenced by the poem's title “Totenklage” (lamentation of the dead) as the only clue to the potential semantic content of the poem.

Additionally to the analysis of literary texts and in a more general context, the presented approach in this article can be used as a complementary method for sentiment classification of written texts. Recent approaches on sentiment and content analysis have awarded much attention to the determination of the contextual polarity of written texts. To this end, different strategies of the Computer-Assisted Text Analysis (CATA) have been applied in an attempt to link word use to the affective state of a given text. These approaches, however, are mostly based on predefined keywords or clusters of keywords and lack a potentially important indicator of a text's emotional state; the sound. On this note, our presented approach can expand upon existing methods for sentiments classification and opinion mining.

In comparison to the analysis of affective and aesthetic effects at the *lexical* level, the *sublexical* level has received less attention by researchers. For instance, in recent years large scale databases for the emotion content of words have been collected (see, for instance, BAWL Võ et al., 2009 for German, and ANEW Bradley and Lang, 1999 for English), and emotion effects during visual word recognition have been reported using a wide range of methodologies: event related potentials (Conrad et al., 2011; Hofmann et al., 2009), hemodynamic responses (Kuchinke et al., 2005, 2006), pupillometry (Võ et al., 2008), and response times (e.g., Kousta et al., 2009; Briesemeister et al., 2011a,b, 2012). Even more recently, also at a *supralexical* level of sentences or larger text passages, emotional and aesthetic effects during reading have been reported (Altmann et al., 2012a,b; Bohrn et al., 2012a,b, 2013; Hsu et al., 2013). The current approach extends this line of research by the focus on such effects at the *sublexical* level. Of course, further research is needed to obtain empirical evidence for the existence of emotional and aesthetic effects of sublexical units at the level of functional neural correlates and substrates.

In concluding, we would like to stress that it is this practical solution of how to define and assess salience that might help theories and hypotheses concerning iconicity or general sound to meaning correspondences in language cross the gap between hermeneutic interpretation and principles of test and falsification widely separating different branches of science involved in the investigation of language and the arts.

### Conflict of interest statement

The authors declare that the research was conducted in the absence of any commercial or financial relationships that could be construed as a potential conflict of interest.

## Footnotes

^1^The splitting was needed because of the limitation of MARY's server performance and its permanent need for online-lookup in lexicon.

## Appendix

### A1. list of poems (poet, title)

1. Stefan George, Auf das Leben und Tod Maximins
2. Christian Morgenstern, Der Papagei
3. Ernst Moritz Arndt, Gottes Scherz
4. Gertrud Kolmar, Die Fremde
5. Heinrich Seidel, Hinter dem Kastanienbaum
6. Johann Gabriel Seidl, Wiederschein
7. Max Dauthendey, Der Himmel ward der Erde gleich
8. Conrad Ferdinand Meyer, In einer Sturmnacht
9. Ernst Moritz Arndt, Mut des Geistes
10. Gottfried Benn, Eingeengt
11. Johann Gabriel Seidl, Ausmarsch
12. Johann Wilhelm Ludwig Gleim, Abschied von Chloris
13. Karl Kraus, In diesem Land
14. Kurt Tucholsky, Vorfruehling
15. Ludwig Anzengruber, Die Herzenskuender
16. Oskar Loerke, Gestaltung
17. Rainer Maria Rilke, Das Fuellhorn
18. Stefan George, Zu meinen traeumen floh ich vor dem volke
19. Heinrich George, Es zuckt aus grauem wolkenzelt
20. Kurt Tucholsky, Deutscher Abend

### A2. list of newspapers' articles (newspapers' name, title hyperlinked)

1. Bild:schwangere-stuttgarterin-setzt-hunde-und-katzen-aus
2. Braunschweiger Zeitung: forscher-praesentieren-meilenstein-beim-klonen
3. Frankfurter Allgemeine Zeitung: bulgarien-wahlsieger-borissow-will-ergebnis-anfechten
4. Frankfurter Rundschau:aerztefehler-keine-entschaedigung-trotz-behandlungsfehler
5. H-N-A:bombendrohung-kasseler-rathaus-arbeitsamt
6. Leipziger Volkszeitung:leipzig-faerbt-sich-schwarz-tausende-pilgern-zum-auftakt-des-wave-gotik-treffens
7. Münchner Merkur:fuerstenfeldbruck-stromausfall-stadtwerke-raetseln-ueber-ursache
8. Mitteldeutsche Zeitung:entlassung-von-wolff-boehmer-kritik-an-haseloff
9. Rheinische Post:zwei-angeklagte-wollen-aussagen
10. Spiegel: silicon-valley-reporter-google-glass-im-kurztest
11. Süddeutsche Zeitung,txt: us-praesident-obama-in-der-kritik-der-enttaeuscher
12. Abendzeitung:malta-oder-san-marino-song-contest-giulia-siegel-blamiert-sich-auf-facebook
13. Berliner Zeitung: suff-pruegler-drischt-auf-busfahrer-ein
14. Göttinger Tageblatt: Warum-der-ICE-an-Goettingen-vorbeigerauscht-ist
15. Hannoversche Allgemeine Zeitung: Wohin-am-Pfingstwochenende
16. Maerkische Allgemeine: Das-Landesverfassungsgericht-verhandelt-heute-die-Klagen-dreier-gut
17. Badische Zeitung: fuerchten-sich-suedbadens-buergermeister-vor-kitaklagen
18. Cellesche Zeitung:Wolf-reisst-Heidschnucke
19. Emder Zeitung: krise-ist-an-dramatik-nicht-zu-ueberbieten
20. Stimme (Heilbronn): Sattelzug-drohte-umzukippen
